# Case Report: Psychoacoustic Analysis of a Clarinet Performance With a Custom-Made Soft Lip Shield Worn to Prevent Mucosal Erosion of Lower Lip

**DOI:** 10.3389/fpsyg.2022.852866

**Published:** 2022-04-21

**Authors:** Gen Tanabe, Mariko Hattori, Satoshi Obata, Yuumi Takahashi, Hiroshi Churei, Akira Nishiyama, Toshiaki Ueno, Yuka I. Sumita

**Affiliations:** ^1^Department of Maxillofacial Prosthetics, Graduate School of Medical and Dental Science, Tokyo Medical and Dental University, Tokyo, Japan; ^2^Department of Sports Medicine/Dentistry, Graduate School of Medical and Dental Science, Tokyo Medical and Dental University, Tokyo, Japan; ^3^Yamaha Corporation, Hamamatsu, Japan; ^4^Department of Oral Diagnosis and General Dentistry, Graduate School of Medical and Dental Science, Tokyo Medical and Dental University, Tokyo, Japan

**Keywords:** lip shield, wind instrument, clarinet, mucosal erosion, performing arts

## Abstract

**Introduction:**

Wind instrument players sometimes suffer from erosion of the mucous membrane of the lip. This is caused by the action and pressure of the mouthpiece of the wind instrument against teeth. To address this problem, a lip shield is fitted over the dental arch to prevent direct contact between the lips and teeth. However, there are a few studies on the influence of the lip shield on the acoustics of wind instruments. The purpose of this study was to analyze the psychoacoustics of a clarinet performance with the player wearing a custom-made soft lip shield to prevent mucosal erosion of the lower lip.

**Case Description:**

A lip shield was custom-made with a soft thermoplastic material for a female clarinetist who complained of mucosal erosion and pain of the lower lip. The psychoacoustics of her musical performance played in different dynamics, fortissimo, mezzo forte and pianissimo were analyzed, including loudness and sharpness. A self-evaluation questionnaire with items rated on a 10-point scale was administered. After wearing the lip shield, the patient reported that the mucosal erosion and pain of her lower lip when playing clarinet resolved. The lip shield had little effect on the loudness. There was a slight decrease in sharpness when the lip shield was worn compared to when it was not, describing the reduction of high frequencies. Furthermore, fewer variations in sharpness between the tones were observed.

**Conclusion:**

The results suggest that lip shields made of soft materials can eliminate mucosal erosion and pain of the lower lip while having little effect on performance, although, a slight change in timbre is possible.

## Introduction

The maxillofacial region is important for sound production, especially when playing wind instruments. In the 1960s, Porter first described the dental aspects of embouchure and conservative tooth treatment for a wind instrument player (Porter, 1967). Porter said that for single-reed instruments, such as clarinets and saxophones, the sharp, chisel-like edges of the incisal enamel tend to cut into the lips in players of these instruments. Often, there is a linear impression on the mucous membrane caused by the teeth, which can be painful and may hinder playing. Thus, wind instrument players sometimes suffer from mucosal erosion of the lip caused by the action and pressure of the wind instrument mouthpiece against teeth. In some instances, it becomes impossible to execute certain specific blowing exercises (Phillips, 1972).

The cause of mucosal erosion and pain of the lower lip in single-reed instrumentalists is thought to be due to the incisal edge morphology of the mandibular anterior teeth and the alignment of the dentition. A recent systematic review stated that tooth position affects the performance and embouchure comfort of wind instrument players, and that extreme malocclusion can interfere with a wind instrumentalist's performance (van der Weijden et al., 2018). Discomfort while playing can also be caused by a combination of other factors, such as the reserve force of the lower lip against mechanical load, that is, the thickness and elasticity of the lower lip, the relationship between the blowing method and playing style, the reed characteristics, time factors, and psychological factors. These factors have not been examined in the literature.

Some wind instrument players have placed folded cigarette-rolling paper or oil-absorbent paper over their lower central incisors while playing to avoid discomfort or pain in the lower lip from playing. In some cases, these solutions may cause musicians to generate greater and unnecessary force in the embouchure, causing discomfort to the teeth and temporomandibular joints after playing (Pais Clemente et al., 2019). Some players have also used commercially available polyolefin resin protectors. These protectors can be easily made by themselves. However, their thickness and design are difficult to control, and they are thought to be less effective, especially when the fit is not good.

A lip shield fitted over the dental arch of the lower jaw to prevent direct contact between the lips and teeth may solve these problems. Lip shields are applied to the mandibular anterior teeth. Materials, such as dental rubber-like materials, silicone rubber, plastic, and metal, have historically been used. Lip shields should be made as thin as possible, covering the incisor, canines, and possibly part of the first premolars (Porter, 1967; Wilson, 1989; Katada et al., 2005). The devices used to protect the lower lip when playing clarinet and/or saxophone have been given different names by different authors, clinicians, or musicians. These devices have been referred to as lip shields, embouchure aids (Krivin and Conforth, 1975), and music splints (Katada et al., 2005; Nii et al., 2021), among others. To date, there is still no consensus regarding the name for lip shields used by instrumentalists. In the present study, with consideration to the historical background, the authors have referred to the device as a lip shield.

In recent years, sports mouthguard materials and fabrication techniques have been applied to lip shields. Sports mouthguard materials are mainly composed of ethylene-vinyl acetate (EVA) and polyolefin, with a Shore A hardness of ~80. Pais Clemente et al. reported the first lip shields made of 1-mm EVA sheets that were stable during a performance and did not interfere with playing (Pais Clemente et al., 2019). Nii et al. (2021) also discussed the optimal stiffness and thickness of EVA sheets and other hard materials based on subjective feedback from instrumentalists and pain resolution. The authors reported that softer and thinner types of lip shields were particularly effective in addressing the pain (Nii et al., 2021). However, few studies have investigated the influence of lip shields made using sports mouthguard materials on the acoustics of wind instrumental performance.

This study aimed to analyze the psychoacoustics of a clarinet performance with the player wearing a custom-made soft lip shield for the prevention of mucosal erosion and pain of the lower lip.

## Case Description

**Figure 1:**
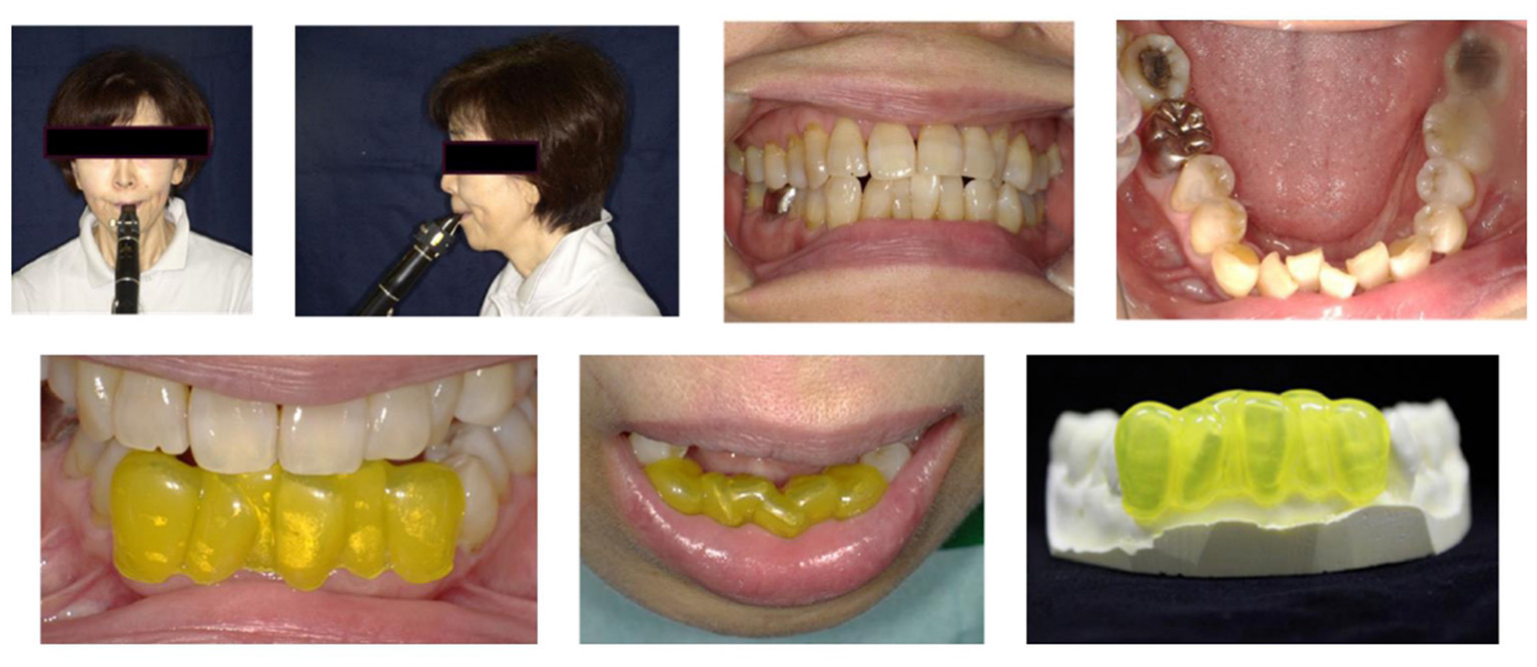
Patient's facial and intraoral photographs and the lip shield.

The patient was a 70-year-old amateur clarinetist. She presented with mucosal erosion and reported pain on the lower lip when playing the clarinet when the duration of practice exceeded 2 h. The duration of daily practice was about 1–2 h. She practices every day and has been playing for about 50 years. At the time of visit, no obvious erosions were seen and there was no color change of mucosa. However, on palpation, a friable lump was felt. The facial and intraoral photographs are shown in Figure 1. The mandibular anterior teeth were plexiform and had chiseled edges. Mesio-distal classification was grade 4, and the bucco-lingual classification was grade 4 according to Chu's classification of dental crowding. The occlusal status of patient was normal occlusion (overbite 0.3 mm, overjet 0.3 mm), and the occlusal supporting area was classified as Eichner's A-1, and there was no tooth movement or defective restoration in this area. We confirmed that the pain in the lower lip occurred where it was caught between the reed and the mandibular anterior teeth when playing the clarinet and inferred that this symptom was due to clarinet playing. The authors think that the prolonged pressure on the lower lip and the resulting blood flow disturbance might be triggering the erosions. Therefore, a lip shield was applied to the mandibular anterior teeth to protect the lower lip from the pressure while playing the clarinet. In this case, the technique and materials of a sports mouthguard were applied to create a lip shield. Soft polyolefin sheets (MG21; CGK, Hiroshima, Japan), 2.0 mm in thickness, were used to fabricate the lip shield.

The lip shield was fabricated as follows. An impression of the mandibular arch was made with an irreversible hydrocolloid (Aroma Fine Plus; GC Corp, Tokyo, Japan). The impression was filled with a dental plaster (New Plastone II; GC Corp., Tokyo, Japan) to make a working cast. The lip shield was fabricated by thermoforming a polyolefin sheet over the working cast using a vacuum forming machine in a vacuum tank (Erkoform 3d motion; Erkodent, Pfalzgrafenweiler, Germany) at 105°C, in the same manner as for the fabrication of sports mouthguards (Tanabe et al., 2020). An image of the design and fabrication of the lip shield is shown in Figure 1. The lip shield covered all six mandibular anterior teeth for enhanced retention. The margins were polished to ensure comfort during clarinet playing. We checked the fit, retention, and occlusion of the lip shield with and without a reed at the dental chair side. The thickness of the buccal lip surface of the lip shield was 1.1 mm. One week after using the lip shield, the patient visited our clinic for follow-up and evaluation.

Her performance was analyzed using psychoacoustic analyses based on loudness and sharpness (Acoustic Workstation SQ6600; Ono Sokki Co, Ltd., Kanagawa, Japan). Psychoacoustic analysis is a sound analysis method based on the characteristics of human audition. In psychoacoustic analysis, loudness and sharpness were the primary outcomes. Loudness is defined as a physical value that represents the sound intensity in human perception (Zwicker et al., 1957). Sharpness is defined as the physical value that represents the balance between low and high frequencies, where high values correspond to higher frequencies of sound (Bismarck, 1974).

**Figure 2:**
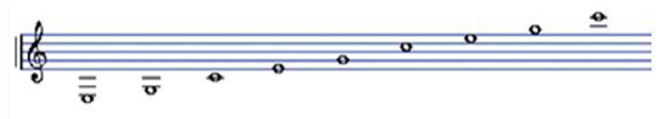
The musical scale task used for psychoacoustic analysis.

Each subject was seated in the chair position and a high-quality dynamic microphone (LA5120; Ono Sokki Co, Ltd, Kanagawa, Japan) was positioned 15 cm away from the instrument. A sound processor (Sound Blaster Extigy; Creative Technology Ltd, Tokyo, Japan) was used, and the recording conditions were 16 bit/96 kHz. Subjects were given an interval of at least 1 s for breath between each test tone. The analysis of each test tone was from the beginning of the tone to the end of the tone. Subjects performed the test only one trial per tone task after one practice session. In task, the following three dynamics were used: pianissimo (very soft), mezzo forte (moderately strong), and fortissimo (very strong). Analyses were performed with and without the lip shield. Figure 2 shows the music-scale task used for psychoacoustic analyses.

And a self-evaluation questionnaire with items on a 10-point scale was administered at 1 week, 3 months, and 8 months to evaluate the usability of the product. The questionnaire rated the following using a 10-point scale: lip shield stability, lip shield discomfort, holding fit, blowing comfort, sound quality, and overall satisfaction, based on Hattori's reports (Hattori et al., 2015).

After wearing the lip shield, the patient reported that the mucosal erosion resolved and the pain of the lower lip she experienced when playing the clarinet also disappeared. The patient wore the lip shield for 1–2 h per day, almost every day. There was no staining or deformation after 8 months of regular use.

**Figure 3:**
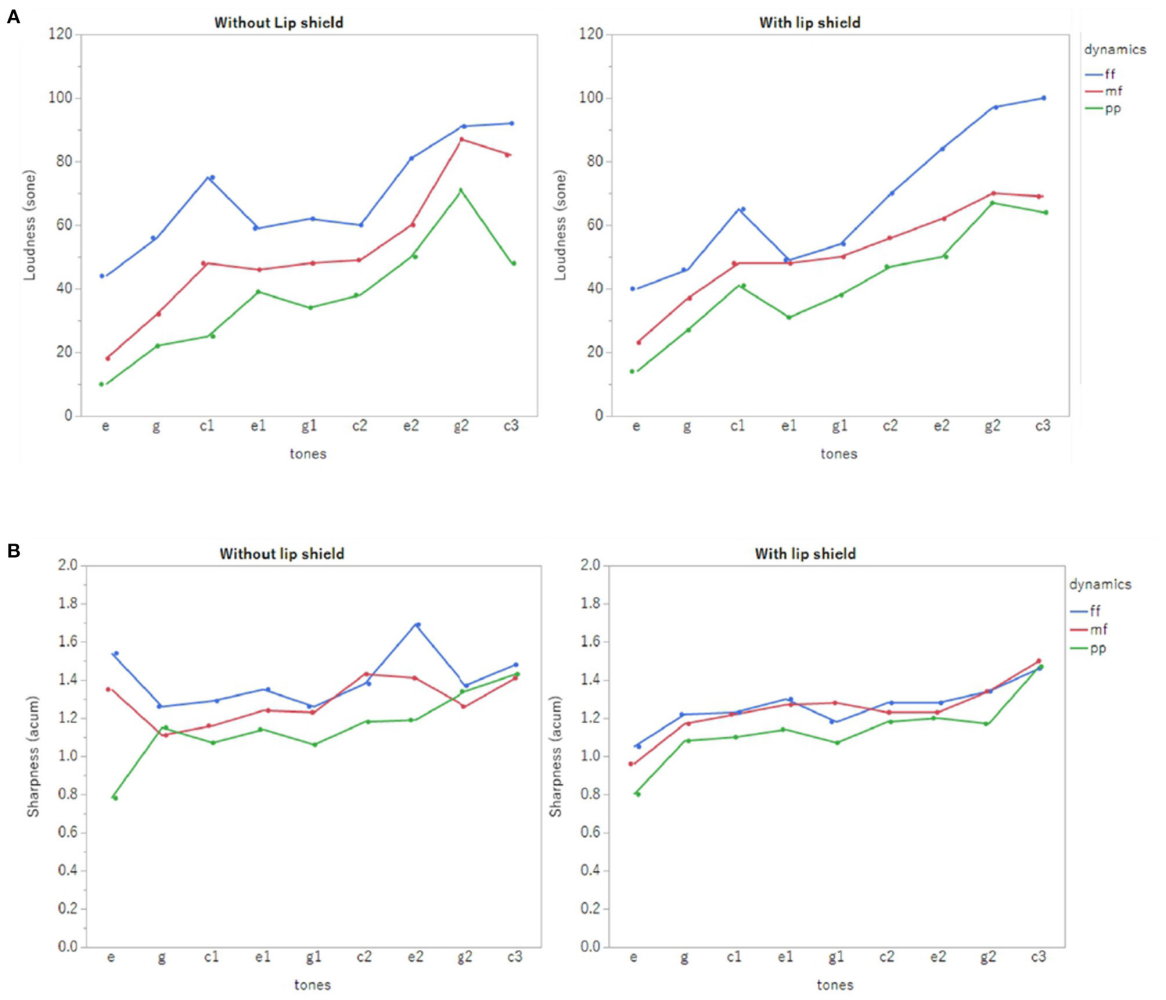
The loudness **(A)** and sharpness **(B)** results of the psychoacoustic analyses.

**Figure 4:**
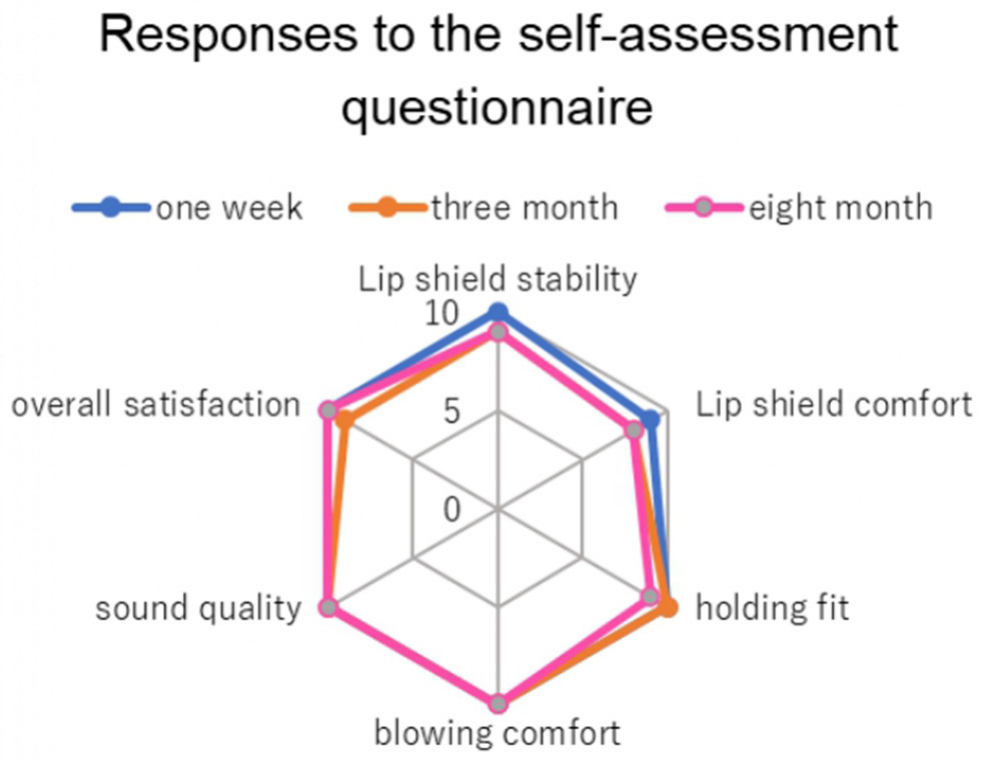
The results of the self-evaluation questionnaire, with items rated using a 10-point scale, at 1 week, 3 months, and 8 months. A score of 10 indicates good and 0 indicates bad.

Figure 3 shows the loudness and sharpness results of the psychoacoustic analyses. The player was able to project the difference of the three dynamics regardless of the use of the lip shield. There was no decrease in loudness when the lip shield was worn, and it was thought that the effect on blowing was small. There was no drastic change in sharpness either with or without lip shields. There was a slight decrease in sharpness when the lip shield was worn compared to when it was not, describing the reduction of high frequencies. Furthermore, there were fewer variations in sharpness between the tones. Figure 4 shows the results of the self-evaluation questionnaire after 1 week, 3 months, and 8 months. All usability outcomes remained favorable over the 8-month follow-up.

## Discussion

In this study, the lip shield prevented pain in the lower lip when playing the clarinet. It is thought that the lip shield buffered the pressure on the lower lip caused by being sandwiched between the incisal edge of the mandibular anterior teeth and the clarinet reed. The effectiveness of the lip shield in preventing pain in the lower lip was similar to that reported in previous studies (Krivin and Conforth, 1975; Nii et al., 2021). A follow-up questionnaire survey of the lip shield's usability over 8 months showed that the product maintained good usability, with no damage or staining observed. These suggested that custom-made soft lip shield was practical as the prevention of mucosal erosion and pain of the lower lip.

Wearing a lip shield over the mandibular anterior teeth is expected to modify the oral volume, exhalation volume, and flow rate of breathing, as well as tongue movements and sensations, thereby changing the overall performance. Because wind instrument players change the dynamics and timbre of their sound by changing the oral capacity, flow rate, and speed of their breathing. Hattori et al. (2014) experimentally altered oral morphology with a palate-covering device and evaluated its performance. Changes in psychoacoustic features and the frequency and intensity ranges were observed. In this study, psychoacoustic analysis and a questionnaire survey were conducted to examine the effects of the lip shield on performance.

Regarding loudness, it was possible to distinguish three different dynamics with or without the lip shield. There was also no decrease in loudness when the lip shield was worn. Since loudness is controlled by the exhalation volume and velocity, the results suggest that lip shields are unlikely to interfere with the exhalation volume and velocity to the extent that they affect performance.

There was a slight decrease in sharpness when the lip shield was worn, and high frequencies were reduced. This change suggests that the metallic component of tone may have changed. The metallic component of the tone is related to the hardness and softness of the sound. The patient in this study reported a change in tone quality, especially a softer sound. It is thought that the lip shield may have changed the vibration applied to the reed during blowing. In particular, the high frequencies were reduced, and the high-frequency components may have been suppressed. This change in vibration is dependent on the way the player holds the reed in the mouth and the material of the reed. There may also be changes depending on the thickness of the lip shield and its fabrication material. It is conceivable that players may adapt to the changes in sound quality caused by this device by changing their technique, such as their embouchure. In such a case, it will be necessary to consider training suggestions for improvement and device design so that these changes do not affect the blowability.

The present study and previous studies provide limited evidence for the validation of the effectiveness of lip shields. In the future, large-scale prospective epidemiological studies and randomized controlled trials, such as those conducted in studies examining the trauma-preventive effects of sports mouthguards, should be conducted to further clarify the effects of lip shields. And the durability of the lip shield and the recommended timing of its replacement needs to be considered in the future. Further clarification of the mechanisms underlying lower lip erosion and pain while playing wind instruments will lead to physically beneficial and improved blowing methods, correct use of lip shields, and better lip shield designs that meet the necessary and desired conditions.

## Conclusion

A lip shield was custom-made with a soft thermoplastic material for a female clarinetist who complained of mucosal erosion and pain of the lower lip. After wearing the lip shield, the patient reported that the mucosal erosion and pain of her lower lip when playing clarinet resolved. The lip shield had little effect on the loudness. There was a slight decrease in sharpness when the lip shield was worn compared to when it was not, describing the reduction of high frequencies. The results suggest that a lip shield made with a soft material can eliminate mucosal erosion and pain in the lower lip, with little effect on performance. Only a slight change in timbre was observed.

## Data Availability Statement

The original contributions presented in the study are included in the article/supplementary material, further inquiries can be directed to the corresponding author/s.

## Ethics Statement

The studies involving human participants were reviewed and approved by the Research Ethics Committee of Tokyo Medical and Dental University (Approval Number: D2016-088). The patients/participants provided their written informed consent to participate in this study. Written informed consent was obtained from the individual(s) for the publication of any potentially identifiable images or data included in this article.

## Author Contributions

GT and YS: conceptualization. GT and MH: methodology and formal analysis. TU: validation. GT, MH, SO, YT, HC, and AN: investigation. GT: data curation, writing—original draft preparation, and funding acquisition. YS and MH: writing—review and editing. TU and YS: supervision. SO: project administration. All authors have read and agreed to the published version of the manuscript.

## Conflict of Interest

SO was employed by Yamaha Corporation. The remaining authors declare that the research was conducted in the absence of any commercial or financial relationships that could be construed as a potential conflict of interest.

## Publisher's Note

All claims expressed in this article are solely those of the authors and do not necessarily represent those of their affiliated organizations, or those of the publisher, the editors and the reviewers. Any product that may be evaluated in this article, or claim that may be made by its manufacturer, is not guaranteed or endorsed by the publisher.
